# NeuroExercise: The Effect of a 12-Month Exercise Intervention on Cognition in Mild Cognitive Impairment—A Multicenter Randomized Controlled Trial

**DOI:** 10.3389/fnagi.2020.621947

**Published:** 2021-01-14

**Authors:** Tim Stuckenschneider, Marit L. Sanders, Kate E. Devenney, Justine A. Aaronson, Vera Abeln, Jurgen A. H. R. Claassen, Emer Guinan, Brian Lawlor, Romain Meeusen, Christian Montag, Marcel G. M. Olde Rikkert, M. Cristina Polidori, Martin Reuter, Ralf-Joachim Schulz, Tobias Vogt, Bernd Weber, Roy P. C. Kessels, Stefan Schneider

**Affiliations:** ^1^Institute of Movement and Neurosciences, German Sport University, Cologne, Germany; ^2^VasoActive Research Group, School of Health and Sport Sciences, University of the Sunshine Coast, Maroochydore, QLD, Australia; ^3^Department of Geriatric Medicine, Radboudumc Alzheimer Center, Radboud University Medical Center, Nijmegen, Netherlands; ^4^Donders Institute for Brain Cognition and Behavior, Nijmegen, Netherlands; ^5^Discipline of Physiotherapy, Trinity College, Dublin, Ireland; ^6^Department of Medical Psychology, Radboud University Medical Center, Nijmegen, Netherlands; ^7^Mercer's Institute for Successful Aging, St. James's Hospital and Global Brain Health Institute, Trinity College, Dublin, Ireland; ^8^Department of Human Physiology & Sports Medicine, Vrije Universiteit Brussel, Brussels, Belgium; ^9^Department of Molecular Psychology, Institute of Psychology and Education, Ulm University, Ulm, Germany; ^10^Aging Clinical Research, Department II of Internal Medicine and Center for Molecular Medicine Cologne, Faculty of Medicine and University Hospital Cologne, University of Cologne, Cologne, Germany; ^11^Center for Economics and Neuroscience, University of Bonn, Bonn, Germany; ^12^Department of Psychology, University of Bonn, Bonn, Germany; ^13^Geriatrics Department, University of Cologne Medical Faculty, Cologne, Germany; ^14^Institute of Professional Sport Education and Sport Qualifications, German Sport University, Cologne, Germany; ^15^Waseda University, Faculty of Sport Sciences, Tokorozawa, Japan

**Keywords:** Alzheimer's disease, non-pharmacological treatment, aerobic exercise, cognition, quality of life

## Abstract

Exercise intervention studies in mild cognitive impairment (MCI), a prodromal stage of Alzheimer's disease (AD), have demonstrated inconsistent yet promising results. Addressing the limitations of previous studies, this trial investigated the effects of a 12-month structured exercise program on the progression of MCI. The NeuroExercise study is a multicenter randomized controlled trial across three European countries (Ireland, Netherlands, Germany). Hundred and eighty-three individuals with amnestic MCI were included and were randomized to a 12-month exercise intervention (3 units of 45 min) of either aerobic exercise (AE; *n* = 60), stretching and toning exercise (ST; *n* = 65) or to a non-exercise control group (CG; *n* = 58). The primary outcome, cognitive performance, was determined by an extensive neuropsychological test battery. For the primary complete case (CC) analyses, between-group differences were analyzed with analysis of covariance under two conditions: (1) the exercise group (EG = combined AE and ST groups) compared to the CG and (2) AE compared to ST. Primary analysis of the full cohort (*n* = 166, 71.5 years; 51.8% females) revealed no between-group differences in composite cognitive score [mean difference (95% CI)], 0.12 [(−0.03, 0.27), *p* = 0.13] or in any cognitive domain or quality of life. VO_2_ peak was significantly higher in the EG compared to the CG after 12 months [−1.76 (−3.39, −0.10), *p* = 0.04]. Comparing the two intervention groups revealed a higher VO_2_peak level in the aerobic exercise compared to the stretching and toning group, but no differences for the other outcomes. A 12-month exercise intervention did not change cognitive performance in individuals with amnestic MCI in comparison to a non-exercise CG. An intervention effect on physical fitness was found, which may be an important moderator for long term disease progression and warrants long-term follow-up investigations.

**Clinical Trial Registration:** https://clinicaltrials.gov/ct2/show/NCT02913053, identifier: NCT02913053.

## Introduction

Worldwide, over 46 million people are living with dementia, with the numbers expected to rise to approximately 74 million by 2030 (Prince et al., 2015). The prevalence of mild cognitive impairment (MCI), a stage of cognitive impairment with minimal functional loss that is often, but not always, a prodromal stage of dementia (McKhann et al., 2011), is 6.7% for ages 60–64 and rises to 25.2% for ages 80–84 (Albert et al., 2011; Petersen et al., 2018). Clinicians and researchers differentiate between amnestic MCI (aMCI), which describes the dominance of memory impairments and is most likely to transition to dementia due to Alzheimer's disease (AD), and non-amnestic MCI, which is characterized by an impairment in other cognitive domains (e.g., language, visuospatial) (Petersen, 2004). Individuals with both aMCI or non-amnestic MCI have a cumulative risk of 14.9% of developing dementia within 2 years (Petersen, 2004; Winblad et al., 2004; Petersen et al., 2018). As such, cognitive decline due to dementia is a key contributor to the significant economic impact of an aging population (Prince et al., 2015), and is identified as a global health and healthcare priority.

If a primary prevention strategy could delay conversion to dementia by even two years, it would reduce the total number of patients living with dementia and have substantial public health, economic and societal benefits (Vickland et al., 2010; Brodaty et al., 2011; Sperling et al., 2011), further highlighting the importance of early detection and treatment of cognitive decline. Currently, there is no proven treatment for people with MCI that delays progression to dementia. However, the recently updated practice guidelines of the American Academy of Neurology (AAN) for the treatment of MCI suggest that exercise is a promising non-pharmacological strategy to improve cognitive function in individuals with MCI (Petersen et al., 2018). This recommendation was underpinned by only two studies that investigated the effect of a 6-month multicomponent exercise or resistance exercise intervention on the progression of MCI and demonstrated a positive effect on domain-specific cognitive function (attention and episodic memory) (Nagamatsu et al., 2012; Suzuki et al., 2012). Furthermore, the results from four systematic reviews recommend exercise as a promising treatment option and included different exercise modes from multicomponent exercises to tai chi in their reviews (Ohman et al., 2014; Wang et al., 2014; Zheng et al., 2016; Song et al., 2018). Whereas three of them recommend aerobic exercise as probably the most effective form of exercise to maintain or improve cognitive function in individuals with MCI (Ohman et al., 2014; Zheng et al., 2016; Song et al., 2018), the other one did not define the type of exercise more specifically (Wang et al., 2014). However, these reviews recommend larger sample sizes, standardized neuropsychological testing, longer intervention periods and well-defined MCI diagnostic criteria, as these were methodological issues limiting previous studies. A recent study by Tarumi and colleagues compared 12 months of either stretching or aerobic exercise training in individuals with aMCI and concluded that both exercise modes improved executive and memory functions (Tarumi et al., 2019). However, the authors did not include a non-exercise control group, which precludes a direct comparison with their trial. Moreover, the two studies included in the AAN guideline included only women or less than 100 participants, which limits their external validity (Nagamatsu et al., 2012; Suzuki et al., 2012).

The multicenter NeuroExercise project addressed these limitations by strictly recruiting participants with aMCI, increasing the sample size compared to previous studies, extending the intervention period, involving three different countries and bringing together experts from clinical and exercise intervention trials (Devenney et al., 2017). The aim of the NeuroExercise project was to investigate the effects of a 12-month structured exercise program (either aerobic exercise or stretching and toning exercises) on the progression of cognitive decline in MCI compared to a control group. We hypothesized that participation in an extensive exercise program, of either aerobic exercise or stretching and toning exercises, would demonstrate a slower rate of cognitive decline compared to the control group.

## Methods

### Trial Overview, Standard Protocol Approvals, and Registrations

The NeuroExercise project was a randomized controlled trial performed in three centers in Europe; the German Sport University Cologne, Germany, Radboud University Medical Center, Nijmegen, the Netherlands and at St. James's Hospital and Trinity College Dublin, Ireland. Participants were randomized to either a yearlong supervised and home-based aerobic exercise program, an equivalent non-aerobic stretching and toning program or to a control group using a centrally controlled computer-generated randomization list (for each country), controlled by an independent statistician.

The ethics committee of the German Sport University, Cologne Germany, the Commisie Mensgebonden Onderzoek Arnhem-Nijmegen, Netherlands, and the Tallaght Hospital/St. James's Hospital Joint Research Ethics Committee Dublin Ireland, approved the study protocol, which has been described previously (Devenney et al., 2017). All participants provided written informed consent to participate in accordance with the provisions of the Declaration of Helsinki. Participants were recruited between October 2015 and September 2017. The trial is registered at Clinicaltrials.gov (trial registration number: NCT02913053).

### Participants and Study Procedure

Sedentary adults aged 50 years or older diagnosed with aMCI were recruited via hospital memory clinics affiliated with the three sites and from the community via advertisements in local newspapers. Eligibility criteria for inclusion were: (1) an education adjusted Montreal Cognitive Assessment (MoCA) score between 18 and 26; (2) stable medical condition for more than 6 months and stable medication for more than 3 months; (3) medical clearance to undergo a symptom-limited cardiopulmonary exercise test and extensive aerobic exercise training; (4) physical ability sufficient to allow performance of endurance exercise training; (5) capacity to provide written and dated informed consent form. Participants recruited from the community completed additional testing to confirm MCI status. To distinguish between amnestic and non-amnestic MCI, we applied education adjusted cut-offs of −2 standard Deviation (SD) for low education (<10 years of education), −1.5 SD for the middle group (10–13 years of education) and −1 SD for the highly educated (>13 years of education), which were taken from the delayed recall portion of either the Logical Memory (story recall) subtest of the Wechsler Memory Scale IV LM (Ireland and Netherlands) or the Repeatable Battery for the Assessment of Neuropsychological Status Delayed Memory Index (Germany) (Randolph et al., 1998; Wechsler, 2008; Hendriks et al., 2014).

Exclusion criteria were: (1) a diagnosis of AD or any other type of dementia; (2) any neurological disorder or other severe chronic disease; (3) engagement in moderate-intensity aerobic exercise training for more than 30 min, three times per week, during past the 2 years. A full list of in- and exclusion criteria as well as sample size calculations have been published previously (Devenney et al., 2017).

### Interventions

In each center participants were randomly assigned to the aerobic exercise (AE), the non-aerobic stretching and toning group (ST) or the control group (CG). Both exercise groups consisted of 3 × 45 min exercise sessions per week over 12 months and exercise intensity was monitored using Borg's Rating of Perceived Exertion (RPE), which is a scale from 6 (“no exertion”) to 20 (“maximal exertion”) that assess subjective perception of effort during exercise (Borg, 1998; Stuckenschneider et al., 2019). Participants of the AE group had a target RPE of at least 13 while exercising, whereas participants in the ST group exercised to an RPE <10 (Borg, 1998). Participants in the AE group performed indoor and outdoor walking and running exercises, whereas participants in the ST group performed light resistance, stretching, and coordination exercises such as balance. Participants attended supervised instructor led classes and completed unsupervised home exercises. Participants were asked to participate in a supervised exercise session at least once a week. Class attendance and adherence to unsupervised home sessions were recorded for all participants. Exercise diaries, which were collected by the class instructors during supervised exercise sessions once a week, were also used to record unsupervised exercise sessions. The CG received usual care and was not advised on exercise or did not attend exercise sessions (Devenney et al., 2017).

### Outcome Measures

All outcomes were measured at baseline (T0) and after 12 months (T2). Outcome assessors were not blinded to the allocated treatment arm. A neuropsychological test battery measuring six cognitive domains (verbal episodic memory, visual episodic memory, working memory, psychomotor function, executive function, and attention) was administered as the primary outcome (Devenney et al., 2017). The neuropsychological test battery consisted of: a computer-based CogState Battery (International Shopping List Task—immediate and delayed recall, Detection Task, Identification Task, One Back Task, and One Card Learning Task), Verbal fluency, and Trail Making Test. The allocation of the tests of the six cognitive domains was based on the CogState Guidelines and conventional classification of neuropsychological tests (Lezak, 2004; Maruff et al., 2004). A comprehensive description of the outcome measures for each test has been published elsewhere (Devenney et al., 2017) and an overview of the domain scores presented in Supplementary Table 1.

Cardiovascular fitness (VO_2_peak) was assessed as a secondary outcome measure using an incremental exercise test on a standard cycle ergometer. Participants at the German Sport University and Trinity College Dublin completed a maximal test in accordance with the World Health Organization Protocol (Fletcher et al., 2001). In Dublin, VO_2_peak was based on direct spirometry [collection of expired gasses during exercise using the K4B2^2^ equipment (K4B2^2^ User Manual, COSMED, Italy)]. In Cologne, VO_2_peak was estimated using the following equation [VO_2_peak = (exercise capacity (W)/weight (kg) × 10.8 + 3.5 + 3.5)] (Glass et al., 2007). In Nijmegen, aerobic fitness was estimated from a submaximal exercise test completed according to the Astrand-Rhyming submaximal protocol, according to the local exercise screening protocol. VO_2_peak was estimated using the average HR of minute 5 and 6 and the work load in the Astrand Nomogram (Astrand and Ryhming, 1954). VO_2_peak (mL/kg/min) was defined as outcome measure for cardiorespiratory fitness.

Health-related quality of life was evaluated using the health-related quality of life for people with Dementia (DemQOL) questionnaire, which has a good acceptability and internal consistency in patients with MCI (Mhaolain et al., 2012). The total score of the DemQOL was calculated and analyzed.

### Statistical Analysis

The primary analysis of this study was the comparison of cognitive functioning (primary outcome measure) before (T0) and after (T2) the 1-year intervention. A composite score was calculated by averaging all six CogState domain scores into one overall cognition score. The obtained scores per test were converted into z-scores based on the standard deviation and mean of the total sample at baseline. In case of multiple tests within one domain, the average score for the domain was calculated with at least one test completed per domain (Supplementary Tables 1, 2). Between-group effect sizes were quantified using Cohen's *d*, with 0.2 representing a small effect, 0.5 a moderate sized effect, and 0.8 representing a large magnitude effect.

For the primary analysis, we included group (aerobic exercise, stretching and toning group, and control group) as independent variables of an ANCOVA with dependent variable the change in composite cognitive score of T2 compared to T0, and as covariates baseline cognitive functioning (T0), sex and age. Between-group differences were analyzed with analysis of covariance under two conditions: (1) the exercise group (EG = combined AE, and ST groups) compared to the control group and (2) AE compared to ST. ANCOVA analyses were chosen as they are unbiased in randomized studies and have more statistical power than repeated measures ANOVAs (Van Breukelen, 2006).

Secondary outcome measures included six separate cognitive domain scores (verbal and visual episodic memory, working memory, psychomotor function, executive function, and attention), cardiorespiratory fitness (VO_2_peak) and quality of life (DemQOL). Analyses for all secondary outcome parameters were carried out with similar ANCOVA analyses using the respective baseline score, age, and gender as covariates. All analyses were performed as complete cases (CC) analyses including all participants independent of adherence to the intervention with baseline and follow-up data of at least one test completed per domain. Data are presented as means and 95% confidence intervals within brackets.

For further exploration of the data and to determine the effect of center, which had a significant influence on recruitment (Sanders et al., 2018), a secondary ANCOVA analysis was performed. Moreover, the effect of per protocol participation on primary outcome measures was included in the secondary analysis. Per-protocol (PP) participation was defined as >66% adherence in the EG, which equaled an average of two exercise sessions per week, in line with recent recommendations from the AAN (Petersen et al., 2018). We included per protocol participation [not per protocol (NPP: ≤66 adherence), per protocol (PP), control group (CG)] and center [Ireland (IRE), the Netherlands (NL), Germany (GER)] as independent variables of an ANCOVA with dependent variable the change in composite cognitive score of T2, and as covariates baseline cognitive functioning (T0), sex and age. Similar secondary analyses were performed for cardiorespiratory fitness (VO_2_peak) and quality of life (DemQOL). In case of significant interaction effects of center^*^per protocol participation, *post-hoc* pairwise comparisons between centers [(IRE, NL, GER) and groups (PP), NPP, CG] were conducted using Bonferroni correction for multiple pairwise comparisons. SPSS 22 was used for all analyses with α set at 0.05.

## Results

### Participants

**Figure 1:**
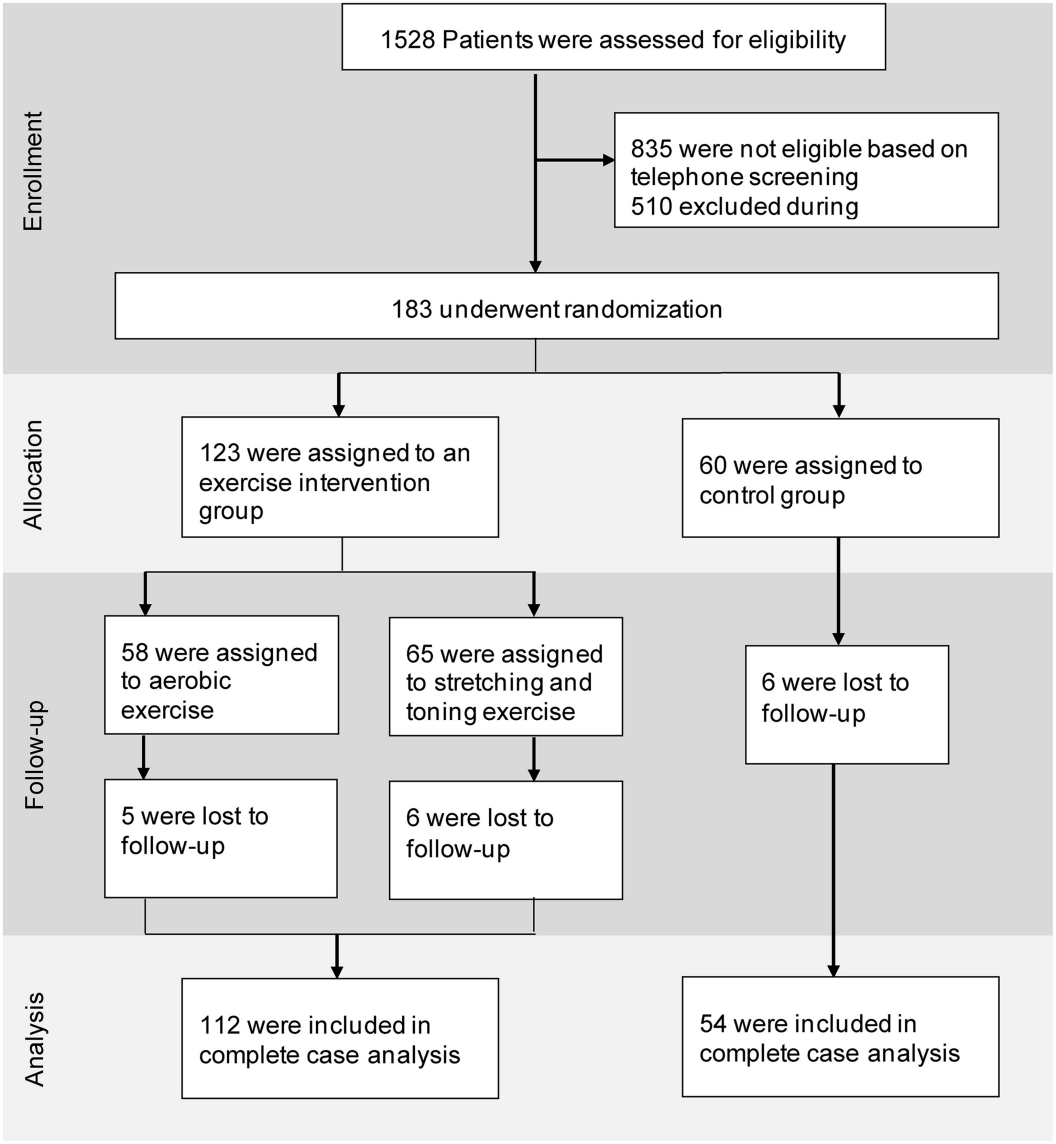
Trial profile.

In total, 183 participants were recruited at the three centers (Germany: 79, Netherlands: 42, Ireland: 62) and randomly stratified into the three groups (AE = 60; ST = 65; CG = 58). Trial recruitment rates differed significantly between the three sites, as discussed previously (Sanders et al., 2018). Five participants dropped out in the AE group (8.6%), six participants in the ST group (9.2%), and six participants in the CG (10%). None of the dropouts were directly related to the intervention, but were due to personal or medical reasons, such as the loss of a relative or diagnosis of cancer. Participant recruitment, screening, enrollment, and attrition are depicted in Figure 1.

Participant characteristics differed between the EG and the CG. The CG had a significantly higher proportion of women and a significantly lower hand grip strength in comparison to the EG (Table 1).

**Table 1:** Group demographics.

	**Exercise (AE + ST)**	**AE**	**ST**	**CG**
	***n* = 125**	***n* = 60**	***n* = 65**	***n* = 58**
Female, *n* (%)	51 (40.8)^*^	28 (46.7)	23 (35.4)	35 (60.3)
Age, mean (SD)	71.5 (6.4)	70.6 (6.1)	72.3 (6.6)	71.6 (6.9)
Center				
IRE	41 (32.8)	18 (30.0)	22 (33.8)	21 (36.2)
NL	32 (25.6)	14 (23.3)	18 (27.7)	10 (17.2)
GER	52 (41.6)	27 (45.0)	25 (38.5)	27 (46.6)
Education, *n* (%)				
Low	8 (6.4)	3 (5.2)	5 (7.7)	6 (10.0)
Middle	56 (44.8)	21 (35.0)	35 (53.8)	33 (56.9)
High	61 (48.8)	36 (60.0)	25 (38.5)	19 (32.8)
MoCA, mean (SD)	22.8 (2.4)	22.6 (2.5)	22.9 (2.2)	22.4 (2.1)
Frailty				
TuG, mean (SD)	8.26 (2.13)	8.06 (1.99)	8.44 (2.25)	8.37 (2.07)
30s, mean (SD)	13.2 (3.7)	13.5 (4.1)	13.0 (3.5)	12.5 (3.2)
Hand grip, mean (SD)	33.5 (11.0)^*^	32.7 (11.1)	34.3 (10.8)	29.4 (10.2)
Exercise sessions. mean (SD)	94.2 (47.0)	96.6 (45.0)	92.1 (48.9)	-
No of medications used. mean (SD)	2.82 (2.75)	2.05 (1.73)	3.54 (3.29)^&^	2.43 (2.17)

*Exercise, both aerobic exercise (AE) and stretching and toning (ST) exercise groups together; CG, control group; IRE, Ireland; NL, Netherlands; GER, Germany; Education categories, low <10 years of education, middle 10–13 years, high >13 years; MoCA, Montreal Cognitive Assessment, education-adjusted score; One-way ANOVA analyses were used to test differences between the exercise group and the control group or the AE and ST comparison*.

* *Significant difference between exercise group and CG, p < 0.05*.

& *Significant difference between AE-ST, p < 0.05. Independent t-test was used to test differences between the number of exercise sessions in AE and ST*.

### Complete Case Analysis

Hundred and twelve participants in the EG (AE = 53; ST = 59) and 54 participants in the CG were included in the CC analysis. Due to missing test results, outcomes have different numbers of cases included (Supplementary Table 2). Individuals in the AE group participated in 96.6 ± 45.0 (mean ± SD) exercise sessions throughout the 12-month intervention period, while participants in the ST group exercised 92.0 ± 49.3 times.

### Complete Case Analysis—Cognition and Quality of Life

**Figure 2:**
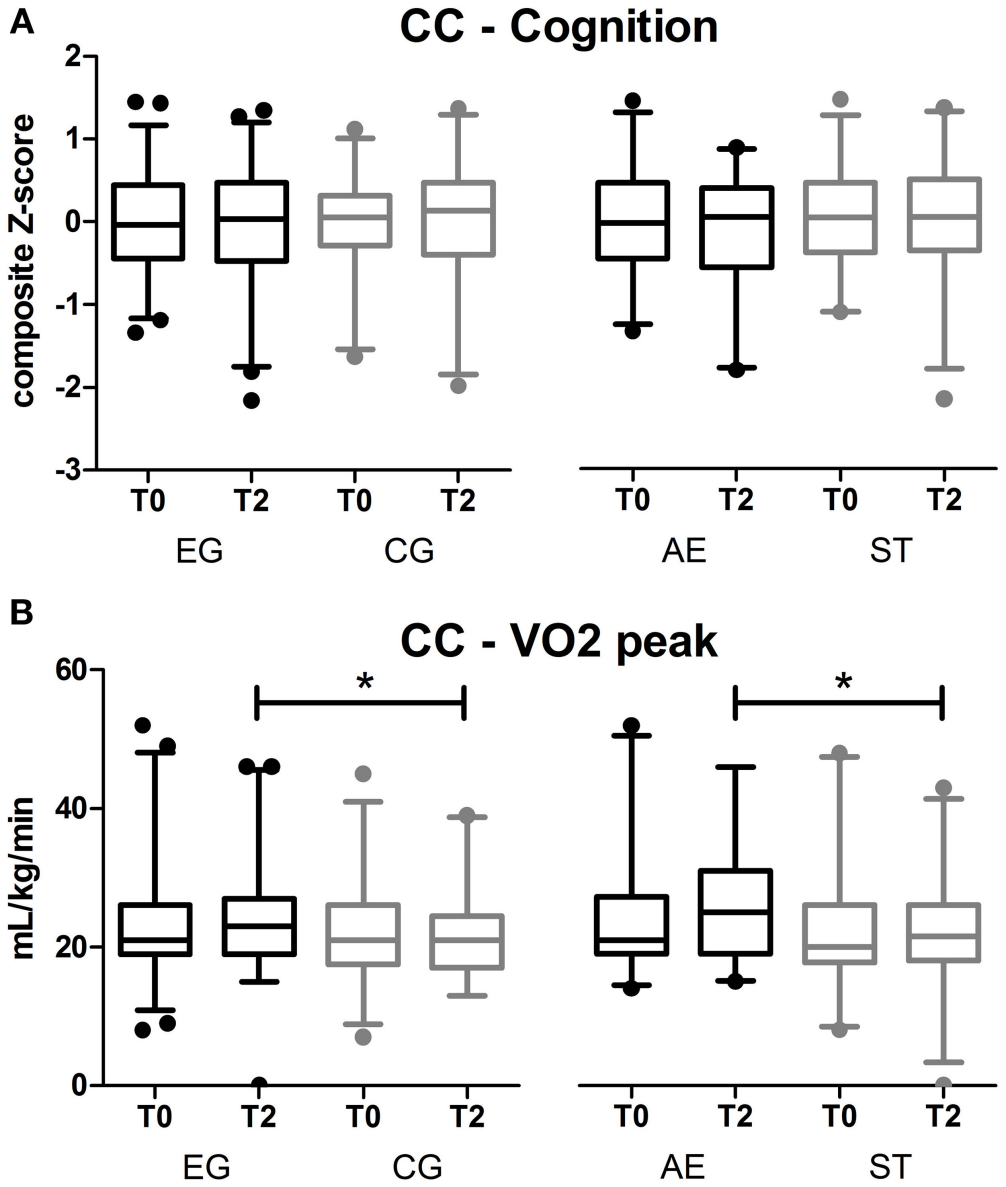
Results of the primary outcome: **(A)** composite cognitive score for complete case (CC) analysis; **(B)** results of VO_2_peak for complete case analysis. Boxplots of mean *z*-scores and 95% CI of EG: Exercise group (AE and ST together). CG, control group; AE, aerobic exercise and ST, stretching and toning exercise. No differences in the comparison between T2 results of the groups for cognition. *significant difference EG vs. CG and AE vs. ST, *p* < 0.05 for VO_2_peak.

ANCOVA did not show a significant difference in composite cognitive performance between the EG and the CG, nor between AE group and ST group, with effect sizes (ES) in the small range, Cohen's d 0.11 [exercise vs. CG, mean difference (95% CI)], [0.12 (−0.03, 0.27)] and 0.22 [AE vs. ST, 0.11 (−0.08, 0.26)] (Figure 2, Supplementary Tables 2, 3). Age (*p* < 0.001), baseline cognitive functioning (*p* < 0.001), but not gender (*p* = 0.45) were associated with T2 cognitive composite performance scores. Furthermore, no significant differences were identified in any of the six cognitive domains nor quality of life between the EG and the CG (Supplementary Tables 2, 3). ANCOVA showed a significant difference between the AE and ST groups for the domain attention [*p* = 0.011 and small ES of 0.35, 0.39 (0.09, 0.67)], where the performance in the ST group was significantly higher compared to the AE group (Supplementary Tables 2, 3).

### Complete Case Analysis—VO_2_Peak

VO_2_peak improved significantly in the EG *p* = 0.04 compared to the CG and in the AE group *p* = 0.001 compared to ST group, with medium ES of 0.40 [−1.76 (−3.39, −0.10)] and 0.60 [−3.10 (−4.95, −1.21)]. ANCOVA revealed that baseline scores were associated with VO_2_peak at T2 (*p* < 0.001), but not age (*p* = 0.88) nor gender (*p* = 0.45).

### Secondary per Protocol and per Center Analysis

**Figure 3:**
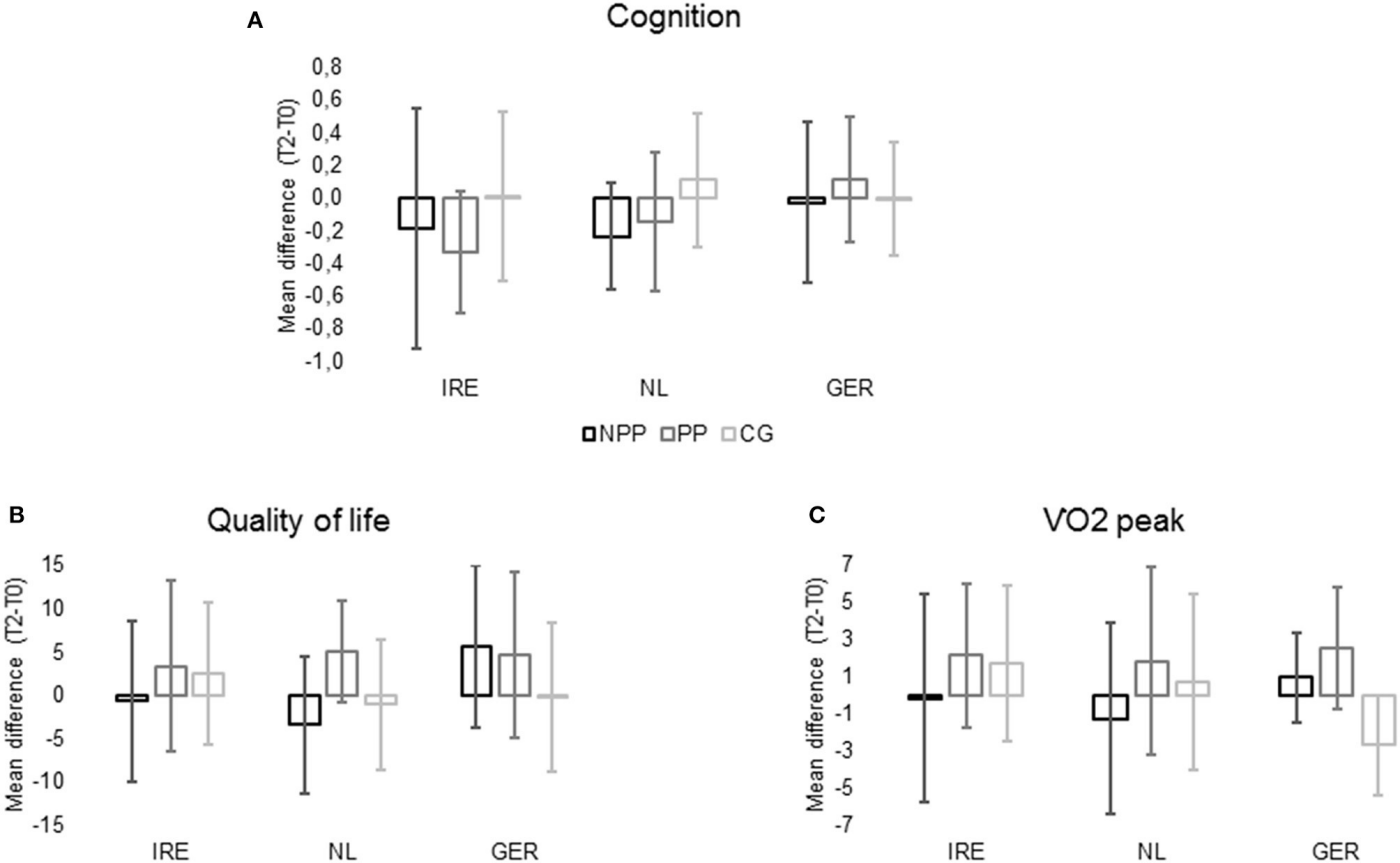
Cognition **(A)** = Mean difference of cognitive composite score between T2 and T0; Quality of Life **(B)** = mean difference of DemQOL total score between T2 and T0; VO_2_peak **(C)** = mean difference of VO_2_peak (ml/kg/min) between T2 and T0 divided by participation and center (NPP, not per protocol; PP, per protocol; CG, control group; IRE, Ireland, NL, Netherlands; GER, Germany; DemQOL, Health-Related Quality of Life for People with Dementia Questionnaire).

One hundred and sixty-six participants (IRE = 56; NL = 36; GER = 74) were included in the secondary analysis. In IRE 19 participants were in the NPP group, 17 participants in the PP group, and 20 participants in the CG. In the Netherlands 8 participants were in each the NPP and the CG, and 20 participants in the PP group. 30 individuals were in the NPP group, 18 in the PP group, and 26 in the CG in Germany. Mean differences for composite cognitive score, quality of life and cardiorespiratory fitness for each group in each center (T2–T0) are presented in Figure 3.

### Secondary Analysis—Cognition

Secondary ANCOVA analysis revealed no effect of group (*p* = 0.069) but an effect of center (*p* = 0.005) on T2 cognitive composite scores. No significant interaction effect between center and per protocol participation for cognitive composite scores (*p* = 0.153) was found. Age (*p* < 0.001) and cognitive functioning at baseline (*p* < 0.001) were associated with T2 cognitive composite scores. No influence of gender (0.673) was identified.

### Secondary Analysis—VO_2_Peak

Per protocol participation (*p* < 0.001), but not center (*p* = 0.772) had an influence on VO_2_peak at T2, and ANCOVA revealed a significant interaction effect of center^*^per protocol participation (*p* = 0.021). *Post hoc* pairwise comparisons showed significant differences for participants in Germany, where individuals in both the PP (*p* = 0.001) and NPP (*p* = 0.019) groups had a significantly higher VO_2_peak compared to participants in the CG. Baseline VO_2_peak (*p* < 0.001), but not gender (*p* = 0.499) or age (*p* = 0.726) were associated with T2 VO_2_peak.

### Secondary Analysis—Quality of Life

No effect of center (*p* = 0.225) or per protocol participation (*p* = 0.051) was found on quality of life. However, a significant interaction effect (center^*^per protocol participation) (*p* = 0.01) was identified. *Post hoc* pairwise comparisons revealed that participants in the NPP group in Germany had a significantly better quality of life in comparisons to participants in IRE (*p* = 0.034) and the NL (*p* = 0.01). Furthermore, in Germany the NPP group had a significantly better quality of life than the CG (*p* = 0.023) at T2. Baseline scores (*p* < 0.001), but not age (*p* = 0.245) or gender (*p* = 0.169) were associated with quality of life after 12 months. No further differences were found in the secondary ANCOVA analysis.

## Discussion

### Main Findings

This multicenter randomized controlled trial analyzed the effects of a 12-month structured exercise program (aerobic exercise or stretching and toning) on the progression of cognitive decline in individuals with aMCI. We did not identify an intervention effect on cognitive performance in the primary complete case analysis. Nevertheless, an intervention effect on physical fitness was identified with a medium effect size. The exercise group increased their physical fitness significantly more after 12 months than the control group.

Collaboration of three research facilities provided the opportunity to analyze aMCI populations across three different countries in North-West Europe. Furthermore, strict inclusion and exclusion criteria ensured a diagnosis of aMCI by using delayed recall scores from standardized memory tests to address limitations of previous studies. An extensive neuropsychological test battery was administered, and composite scores calculated to provide insight into both general cognitive performance and domain-specific cognitive function before and after participation.

Recently, two independent systematic reviews (Zheng et al., 2016; Song et al., 2018) as well as the American Academy of Neurology (AAN) (Petersen et al., 2018) suggested a positive effect of exercise on cognitive function in individuals with MCI. However, the results of our study do not corroborate this, as neither the EG nor the CG improved or decreased their cognitive performance over the period of 12 months. Even though it can be argued that a stable cognitive function may be positive for individuals with MCI, other studies demonstrated improvements on specific cognitive tests after an exercise intervention (Nagamatsu et al., 2012; Suzuki et al., 2013; Nascimento et al., 2014; Tarumi et al., 2019). In contrast to previous studies, we used composite outcome measures for different cognitive domains based on standardized neuropsychological tests, which is considered the best approach to detect cognitive changes in individuals at risk of AD (Lim et al., 2016).

To date, it is unclear which people with MCI progress to dementia, remain stable, or reverse to normal cognitive function and studies report different numbers that may explain outcomes of our study. The AAN summarized findings of different studies and calculated a cumulative risk of 14.9% for the development of dementia in individuals with MCI within two years (Petersen et al., 2018). However, other studies also showed a reversion to unimpaired cognitive function on their follow-up measurements of their respective participants (Ganguli et al., 2011; Roberts et al., 2014). Individuals with aMCI are reportedly at a higher risk of progressing to dementia (Albert et al., 2011; Ganguli et al., 2011; Roberts et al., 2014), but over the course of 12 months stability in cognitive function may be the most frequently observed outcome (Ganguli et al., 2011). Ganguli and colleagues reported a progression to dementia of only 1.4% for individuals with aMCI with 77.8% remaining stable and further 15.4% improving their cognitive function back to normal after 12 months (Ganguli et al., 2011). Therefore, mixed outcomes in different studies may be expected in a cohort of individuals with any type of MCI.

MCI does not have one single cause, but is a multicausal syndrome (Knopman and Petersen, 2014; Petersen et al., 2014), which might explain the different outcomes observed in our and other studies as it is unlikely that one single treatment (e.g., exercise) will prove to be an effective intervention for all individuals. Multidomain-type (e.g., diet, exercise, and cognitive training) interventions in the Finnish Geriatric Intervention Study to Prevent Cognitive Impairment and Disability (FINGER) showed positive results on global cognitive function, as well as on processing speed and executive function in a sample of older individuals at cerebrovascular risk for cognitive decline (Ngandu et al., 2015). However, the FINGER study showed this significant treatment benefit in a cognitively unimpaired sample of older adults at risk for future cognitive decline, but not in individuals with diagnosed MCI (Ngandu et al., 2015).

Despite the lack of significant differences in cognitive function, physical fitness—measured by VO_2_peak—increased significantly in the EG compared with the CG, and in the AE group compared with the ST group. These findings are in line with existing literature, as standardized exercise training increases physical fitness (Tarumi et al., 2019). Even though a direct effect of an increased VO_2_peak on cognition was not detected, higher physical fitness might be an important outcome for future disease progression. Recently published research defined changes in physical fitness as an independent risk factor for incident dementia and dementia mortality and highlighted the importance of improving fitness to delay onset of dementia (Tari et al., 2019). This is in line with previous research, which showed that higher physical fitness during mid-life and late-life (e.g., higher VO_2_peak values) is positively associated with cognitive function in older adults with and without cognitive impairment (Mavros et al., 2017; Schultz et al., 2017; Stuckenschneider et al., 2018). Individuals with MCI have an increased risk of being socially isolated, which increases the risk of future progression to dementia (Fratiglioni et al., 2000; Bosma et al., 2002). Previous studies showed that higher physical fitness leads to an increase in self-confidence, which consequently may increase social participation (Perkins et al., 2008; Choi et al., 2017). Therefore, improving physical fitness may not only benefit cognitive function, but also may be the driving force for a socially integrated and fulfilling life during later life—especially in individuals with MCI. However, further evidence (e.g., longer intervention periods, longer follow-up period) is required to establish the beneficial effects of an increased VO_2_peak on cognitive decline.

Moreover, increased fitness likely induces structural changes such as an increased hippocampal volume and an improved white matter integrity (Young et al., 2015; Dougherty et al., 2017; Muller et al., 2017; Ding et al., 2018). Findings to date are equivocal if exercise induced structural changes influence cognitive function directly or if structural changes may rather be beneficial for sustaining cognitive functions long-term (Hotting and Roder, 2013). To further explore this hypothesis, data from physiological measurements (e.g., MRI scans) is needed to provide insight into physiological mechanisms triggered by an increased physical fitness. Tarumi and colleagues provided insight into possible mechanisms and showed that an increased cardiorespiratory fitness following a 12 months exercise intervention was associated with an improved white matter integrity of the prefrontal cortex (Tarumi et al., 2019, 2020) as well as a redistribution of cerebral blood flow in individuals with aMCI (Thomas et al., 2020). However, the lack of a control group as well as an overall atrophy of global brain volume and particularly hippocampal areas warrants further research (Tarumi et al., 2019).

While no effective treatment currently exists for AD, a large number of mechanisms related to AD genetics and different modifiable risk factors, as well as protective factors (such as exercise) have been identified. Given the large heterogeneity in current studies, it may be time to rethink future trials whereby personalized precision prevention may be the most appropriate approach, in which an intervention is prescribed in response to each individuals' modifiable risk factors (Gillman and Hammond, 2016). A possible next step towardz this may include a responder analysis to identify which individuals with MCI benefit most from an exercise intervention (Snapinn and Jiang, 2007). Further research is needed to better define the biomarkers or cognitive profile that best predicts different subtypes of MCI, especially those at the highest risk to progress from the preclinical stages of MCI towards dementia due to AD (Sperling et al., 2011).

### Secondary Analysis—Effect of Research Setting

In none of the centers cognitive function significantly improved in the exercise groups at T2. However, participants in Germany exercising at least twice a week were the only participants in the exercise groups that showed a positive trend on their cognitive function, based on a positive difference between T2 and T0. Additionally, physical fitness and quality of life significantly improved in the exercise groups in comparison to the control group in Germany, whereas no significant changes for any outcome in Ireland and the Netherlands were found. Therefore, we speculate that a non-medical setting and a non-medical research community might have additional effects on improving fitness and may have additional benefits for quality of life in individuals with MCI (Sanders et al., 2018). Even though multicenter clinical studies in elderly with cognitive impairment are the backbone of evidence-based prevention, complete standardization is difficult to obtain in the different research facilities involved. Whereas the exercise classes in Germany were all supervised, participants in Ireland and the Netherlands partly exercised on their own. Even though all participants were instructed on exercise duration and intensity and were asked to participate in at least one supervised exercise session a week to remind and educate them about exercise intensity (Devenney et al., 2017), unsupervised exercise training may compromise treatment fidelity. Further, it is possible that methods such as self-report of exercise training are open to bias due to the so-called social desirability response bias (Aadahl and Jorgensen, 2003), which may explain findings of the secondary analysis with physical fitness only improving significantly in Germany. Besides the additional effects of supervised exercise training on VO_2_peak, increased social interaction, which was achieved due to the participants exercising in groups during the supervised sessions, may also explain secondary findings for quality of life, which only improved in the German sample. However, we did not find a significant treatment effect on cognition for the German sample.

### Strength and Limitations

This study has several strengths including its large sample size, its strict inclusion of individuals with aMCI, its long intervention period (12 months), and its multicentric design. Furthermore, different cognitive tests were combined into one overall score of cognitive function (primary outcome), but also for different subdomains of cognitive function to provide a psychometrically more reliable and thus more valid construct (Lim et al., 2016). However, subtle changes may be easier detected in single tests—even though they are also prone to false positive type I errors.

One of the limitations of the study was that the recruitment aim of 225 individuals with aMCI (75 per center), was not achieved. Nevertheless, recruitment numbers were sufficient regarding the power calculation for primary analysis (16). As numbers were significantly lower in the secondary analysis, due to the differentiation by center, results of it need to be interpreted cautiously. Despite difficulties in recruitment, dropout rates were less than 10% in all groups, which shows a good acceptance of the study and the high motivation of the participants. Participants received their individual study results after completion of the study, which may have motivated individuals to participate, especially in the CG. As cardiorespiratory fitness did not change in this CG, it is unlikely that the participants in that group as a whole altered their lifestyle only because of participating in our trail.

Only 53% of participants reached the target exercise frequency, which was defined as 100 exercise sessions (or more, at least twice a week) within the intervention period of 12 months according to recent recommendations of the AAN (Petersen et al., 2018). In comparison to previous studies the low number of participants following the per-protocol intervention may have been due to the longer intervention period (12 months), and the strict and conservative target exercise frequency. The low dropout rate and more than half of the intervention group following the strict protocol is already a promising result for this rather inactive participant selection, which is reflected by a low baseline cardiorespiratory fitness in comparison to population reference values (Edvardsen et al., 2013). As the best dose-response relationship of exercise on cognitive function is frequently discussed, yet still unknown (Gomes-Osman et al., 2018), future exercise prevention trials should concentrate on defining the best dose-response relationship. Future studies should use objective monitors such as heart rate monitors to ensure a certain exercise intensity is reached. Even though Borg's RPE scale may present a practical tool to monitor intensity, self-reporting always bears the risks to be affected by motivational aspects and over- or underestimation of the individuals' functional abilities. Therefore, monitoring intensity via heart rate may increase accuracy of the training program and, thus, lead to better study outcomes. It may be speculated that different outcomes observed in our study compared to the one conducted by Tarumi and colleagues may be due to the objective monitoring used in their study (Tarumi et al., 2019). However, cardiorespiratory fitness increased in both trials, so that further insight into these different monitoring methods is warranted.

Different tests for VO_2_peak were applied, which was due to different regulations by the institutional review boards (Devenney et al., 2017). Effects of these differences were minimized by using standardized and validated VO_2_peak measurements at all sites. Significant differences between the groups' baseline characteristics are reported, however, these occurred rather by chance than by bias (Altman and Dore, 1990; Moher et al., 2010), as a centrally controlled computer-generated randomization list (for each country), which was controlled by an independent statistician, was used. There is ongoing discussion whether to report differences in baseline characteristics or not (Moher et al., 2010). Given the large heterogeneity observed in individuals with MCI, we presented baseline statistics to identify possible confounders. The EG and CG had a different proportion of women and men with significantly more women being in the CG. This could have biased the results, as women have a higher risk to progress to dementia, but we performed an ANCOVA to statistically adjust for the potential effect of sex. The different proportion of women and men might also account for the differences in hand grip strength between the EG and the CG. As men are reportedly stronger than women, a higher value in hand grip may be expected. Furthermore, the ST group used significantly more medications compared to the AE group. As investigational drug studies and unstable medication were among the exclusion criteria of our trial, it is unlikely that this baseline difference had a significant impact on our results. However, the effect of medication has not fully been investigated in previous studies on exercise interventions and should be addressed by future trials.

### Future Directions

No cure for AD currently exits, prompting increased efforts to understand the preclinical stages such as aMCI as potential opportunities for new treatment approaches. Based on results of our multicentric randomized controlled trial, future research in the field of exercise interventions should target the following aspects: Both cognitive and physiological measurements are warranted to fully establish the effects of exercise and provide insight into its underlying mechanisms. Furthermore, different and combined exercise modes (e.g., endurance, resistance), intensities (e.g., moderate, high) and exercise volume (length of exercise classes, average exercise sessions per week) need attention to identify best dose-response relationships in the future. To ensure comparability between studies researches must focus on uniform reporting of their findings and their interventions. Objective monitors such as heart rate/activity monitors should be used to measure exercise intensity and frequency. Additionally, long-term follow-ups are needed to establish the long-term effects of exercise on disease progression. Besides promoting exercise on its own, multi-domain type interventions (e.g., exercise, cognitive training, and diet) may help to address the large heterogeneity observed in individuals in preclinical stages of AD.

## Conclusion

This study does not support the recommendation from small and short-term RCTs that an exercise intervention has an effect on cognitive performance in individuals with amnestic MCI. Nevertheless, we found a reliable intervention effect on physical fitness, which may be an important outcome for disease progression. Future trials need to target long-term follow ups (up to 5 years) to evaluate the efficacy of an increased physical fitness on cognitive decline. Moreover, the heterogeneity between subjects and centers may explain different findings within the study. Therefore, future trials should consider personalized intervention approaches or multidomain interventions.

## Data Availability Statement

The datasets used and analyzed during the current study will be made available by the corresponding author upon reasonable request.

## Ethics Statement

The studies involving human participants were reviewed and approved by the Ethics Committee of the German Sport University, Cologne Germany, the Commisie Mensgebonden Onderzoek Arnhem-Nijmegen, Netherlands, and the Tallaght Hospital/St. James's Hospital Joint Research Ethics Committee Dublin Ireland approved the study protocol. The patients/participants provided their written informed consent to participate in this study.

## Author Contributions

SS, BL, MO, MP, R-JS, CM, MR, BW, and RM were responsible for conceptualization. TS, MS, and KD carried out data curation were responsible for the investigation. Further, TS, MS, and RK performed the formal analysis. Funding was acquired and resources and supervision were provided by SS, BL, and MO. SS, VA, TV, TS, RK, MO, JA, MS, BL, EG, and KD designed the methodology of the study. SS, BL, MO, TS, MS, and KD were responsible for the project administration. TS and MS wrote the original draft of the manuscript and were responsible for visualization, while RK, KD, MO, BL, SS, MP, VA, and EG reviewed and edited the manuscript. Validation of the analysis and the data was provided by MS, MO, RK, MR, and SS. All authors have read and approved the final version of the manuscript, and agree with the order of presentation of the authors.

## Conflict of Interest

The authors declare that the research was conducted in the absence of any commercial or financial relationships that could be construed as a potential conflict of interest.
