# Supplementary material for: NeuroExercise: The Effect of a 12-Month Exercise Intervention on Cognition in Mild Cognitive Impairment—A Multicenter Randomized Controlled Trial
Source: Front Aging Neurosci. 2021 Jan 14;12:621947. doi: 10.3389/fnagi.2020.621947 (PMC7840533; doi:10.3389/fnagi.2020.621947)
Supplement: Supplementary file 1 [file Table_1.DOCX]

| **Table S1: Detailed overview of the cognitive test battery** | | | |
| --- | --- | --- | --- |
| **Domain** | **Test** | **Unit** | **Reversed score** |
| Verbal memory | International shopping list | Number of correct responses | -  - |
|  | International shopping list delayed recall | Number of correct responses |  |
| Psychomotor function | Detection Task | Accuracy/Reaction time in ms | - |
| Executive function | Trail Making Test B/Trail Making Test A | Time in s | Yes |
|  | Letter Fluency | Number of correct responses | - |
|  | Category Fluency | Number of correct responses | - |
| Attention | Identification Task | Accuracy/Reaction time in ms | - |
|  | Trail Making Test A | Time in s | Yes |
| Working memory | One Back Task | Accuracy | - |
| Visual memory | One Card Learning Task | Accuracy | - |

**Supplementary tables**

The tasks international shopping list (direct and delayed recall), detection task, identification task, one back task, and one back learning task are part of a computer based CogState Battery (<https://cogstate.com>). The Trail Making Test A + B, as well as letter and category fluency tasks are paper and pencil tests. Score is reversed if a lower score = better performance
