# Supplementary material for: NeuroExercise: The Effect of a 12-Month Exercise Intervention on Cognition in Mild Cognitive Impairment—A Multicenter Randomized Controlled Trial
Source: Front Aging Neurosci. 2021 Jan 14;12:621947. doi: 10.3389/fnagi.2020.621947 (PMC7840533; doi:10.3389/fnagi.2020.621947)
Supplement: Supplementary file 2 [file Table_2.DOCX]

**Supplementary tables**

| **Table S2: Results of complete case analysis** | | | | | | | | | | | | | | | | | | | | | | | | |
| --- | --- | --- | --- | --- | --- | --- | --- | --- | --- | --- | --- | --- | --- | --- | --- | --- | --- | --- | --- | --- | --- | --- | --- | --- |
|  | **Exercise (AE + ST)** | | | | | | **Aerobic training** | | | | | | **Stretching and toning training** | | | | | | **Control group** | | | | | |
|  | **T0** | | | **T2** | | | **T0** | | | **T2** | | | **T0** | | | **T2** | | | **T0** | | | **T2** | | |
|  | **N** | **Mean** | **SD** | **N** | **Mean** | **SD** | **N** | **Mean** | **SD** | **N** | **Mean** | **SD** | **N** | **Mean** | **SD** | **N** | **Mean** | **SD** | **N** | **Mean** | **SD** | **N** | **Mean** | **SD** |
| **CC results** |  |  |  |  |  |  |  |  |  |  |  |  |  |  |  |  |  |  |  |  |  |  |  |  |
| **Cognition composite** | 125 | 0.01 | 0.59 | 113 | -0.09 | 0.74 | 60 | -0.01 | 0.62 | 54 | -0.15 | 0.74 | 65 | 0.03 | 0.56 | 59 | -0.02 | 0.74 | 58 | -0.01 | 0.53 | 53 | -0.01 | 0.69 |
| **V̇O_2_peak (mL/kg/min)** | 116 | 23.21 | 7.78 | 98 | 24.15 | 8.13 | 58 | 24.21 | 8.21 | 47 | 26.33 | 8.31 | 58 | 22.21 | 7.25 | 51 | 22.14 | 7.49 | 57 | 21.63 | 6.42 | 49 | 21.32 | 5.68 |
| **DemQOL (total score)** | 124 | 89.25 | 11.51 | 111 | 92.59 | 10.97 | 60 | 88.42 | 12.44 | 54 | 93.19 | 11.29 | 64 | 90.03 | 10.61 | 57 | 92.02 | 10.72 | 57 | 91.75 | 10.83 | 52 | 92.06 | 11.79 |
| **No of exercise sessions** | 121 | 94.25 | 46.95 |  |  |  | 57 | 96.63 | 44.99 |  |  |  | 64 | 92.13 | 48.89 |  |  |  |  |  |  |  |  |  |
| **Visual episodic memory** | 124 | -0.02 | 1.03 | 111 | -0.02 | 1.08 | 59 | -0.17 | 1.03 | 54 | -0.17 | 1.11 | 65 | 0.11 | 1.02 | 57 | 0.12 | 1.03 | 58 | 0.05 | 0.94 | 53 | 0.19 | 1.06 |
| **Verbal episodic memory** | 124 | -0.05 | 0.95 | 112 | 0.04 | 1.08 | 59 | -0.11 | 1.04 | 54 | 0.03 | 1.18 | 65 | 0.00 | 0.87 | 58 | 0.04 | 0.99 | 58 | 0.12 | 0.94 | 53 | 0.12 | 1.04 |
| **Working memory** | 124 | 0.02 | 0.98 | 111 | 0.03 | 1.20 | 59 | 0.06 | 0.96 | 53 | -0.02 | 1.17 | 65 | -0.01 | 1.00 | 58 | 0.08 | 1.23 | 58 | -0.05 | 1.04 | 52 | 0.04 | 1.16 |
| **Attention** | 125 | 0.00 | 0.77 | 113 | -0.22 | 1.08 | 60 | 0.02 | 0.78 | 54 | -0.37 | 1.17 | 65 | -0.02 | 0.76 | 59 | -0.08 | 0.97 | 58 | 0.01 | 0.82 | 53 | -0.11 | 0.81 |
| **Executive** | 125 | 0.01 | 0.77 | 113 | 0.17 | 0.78 | 60 | -0.05 | 0.82 | 54 | 0.09 | 0.79 | 65 | 0.08 | 0.71 | 59 | 0.23 | 0.76 | 58 | -0.04 | 0.66 | 53 | 0.11 | 0.66 |
| **Psychomotor** | 123 | 0.07 | 0.97 | 112 | -0.45 | 0.98 | 59 | 0.12 | 1.00 | 54 | -0.48 | 0.90 | 64 | 0.01 | 0.95 | 58 | -0.42 | 1.05 | 58 | -0.14 | 1.05 | 53 | -0.38 | 1.06 |

**(AE= aerobic exercise; ST = stretching and toning training; SD =standard deviation, DemQOL = Health-Related Quality of Life for People with Dementia Questionnaire; for cognition composite and the six different cognitive domains higher scores reflect a better performance)**
