# Supplementary material for: NeuroExercise: The Effect of a 12-Month Exercise Intervention on Cognition in Mild Cognitive Impairment—A Multicenter Randomized Controlled Trial
Source: Front Aging Neurosci. 2021 Jan 14;12:621947. doi: 10.3389/fnagi.2020.621947 (PMC7840533; doi:10.3389/fnagi.2020.621947)
Supplement: Supplementary file 3 [file Table_3.DOCX]

| **Table S3: Results of the ANCOVA for the complete case**  **analysis** | | | | | | | | | | |
| --- | --- | --- | --- | --- | --- | --- | --- | --- | --- | --- |
|  | **Comparison between exercise (AE and ST) and CG** | | | | | **Comparison between AE and ST** | | | | |
|  |  | **Effect size** |  | **Confidence interval 95%** | |  | **Effect size** |  | **Confidence interval 95%** | |
|  | **p – value** | **Cohens-d** | **mean diff** | **lower** | **upper** | **p - value** | **Cohens-d** | **mean diff** | **lower** | **upper** |
|  |  |  |  |  |  |  |  |  |  |  |
| **Cognition composite** | 0.12 | 0.11 | 0.12 | -0.03 | 0.27 | 0.31 | 0.22 | 0.11 | -0.08 | 0.26 |
| **V̇O_2_peak** | 0.04 | 0.40 | -1.76 | -3.39 | -0.10 | 0.01 | 0.60 | -3.10 | -4.95 | -1.21 |
| **DEMQOL** | 0.21 | 0.02 | -1.89 | -4.59 | 1.00 | 0.31 | 0.11 | -1.51 | -4.71 | 1.51 |
| **Visual episodic memory** | 0.29 | 0.21 | 0.17 | -0.14 | 0.47 | 0.53 | 0.31 | 0.16 | -0.24 | 0.46 |
| **Verbal episodic memory** | 0.98 | 0.04 | -0.01 | -0.23 | 0.23 | 0.61 | 0.01 | -0.04 | -0.33 | 0.19 |
| **Working memory** | 0.82 | 0.02 | 0.04 | -0.30 | 0.38 | 0.48 | 0.2 | 0.17 | -0.25 | 0.52 |
| **Attention** | 0.25 | 0.10 | 0.14 | -0.11 | 0.41 | 0.01 | 0.3 | 0.39 | 0.09 | 0.67 |
| **Executive** | 0.83 | 0.05 | 0.03 | -0.14 | 0.18 | 0.74 | 0.2 | 0.03 | -0.15 | 0.21 |
| **Psychomotor** | 0.13 | 0.07 | 0.24 | -0.07 | 0.53 | 0.48 | 0.07 | 0.15 | -0.21 | 0.46 |

**Supplementary tables**

**(AE= aerobic exercise; ST = stretching and toning training; SD =standard deviation)**
